# mGem: Subcellular compartments in bacterial pathogens and their role during infection

**DOI:** 10.1128/mbio.03328-25

**Published:** 2026-07-01

**Authors:** Christopher J. Corcoran, Eric P. Skaar

**Affiliations:** 1 Department of Pathology, Microbiology, and Immunology, Vanderbilt Institute for Infection, Immunology, and Inflammation, Vanderbilt University Medical Center 12328, Nashville, Tennessee, USA; Georgia Institute of Technology, Atlanta, Georgia, USA

**Keywords:** subcellular compartments, bacterial pathogenesis, encapsulin, bacterial microcompartment, ferrosome

## Abstract

Bacterial subcellular compartments and organelles allow for the spatial organization and sequestration of metabolic pathways, toxic intermediates, and key nutrients. The prevalence of these compartments across bacterial phyla, including in prominent human pathogens, has become increasingly defined in the literature. However, the functions of subcellular compartments in bacterial pathogens and their contributions to virulence remain understudied, leaving many questions unanswered. In this review, we summarize current findings regarding the prevalence of proteinaceous and membrane-bound compartments in pathogenic bacterial species and their role during infection, and we highlight important aspects where new discoveries could have the greatest impact. Further insight into the function of these subcellular structures may reveal novel targets for antimicrobial development against the increasing threat of multi-drug-resistant pathogens.

## PERSPECTIVE

Proteinaceous or membrane-bound organelles allow for discrete sequestration of labile or toxic metabolites, concentration of enzymatic substrates and pathways, or storage of key nutrients within the cell cytoplasm (1). In contrast to eukaryotes, subcellular organization and compartmentalization strategies in bacteria are relatively recent discoveries. Advanced imaging technologies and bioinformatic approaches to mine genomic databases have led to a marked increase in the number of identified subcellular compartments in bacteria (2–4). It is now appreciated that subcellular compartments are ubiquitous among bacteria, with structures identified across gram-negative and gram-positive organisms and spanning a wide range of bacterial phyla. The distinct functions of these compartments can vary widely across bacterial species, with species- or genera-specific compartments expressed in response to external stimuli or changes in the environment (1). These include compartments involved in navigation (magnetosomes) (5), light harvesting (chlorosomes) (6), iron storage (ferrosomes) (7), carbon fixation (carboxysomes) (8), and nutrient utilization and storage (bacterial microcompartments and acidocalcisomes, respectively) (9, 10).

Many of these specialized compartments, such as chlorosomes and carboxysomes, have only been identified in non-pathogenic bacteria, as they are utilized to harvest light or fix carbon dioxide in cyanobacteria and other photoautotrophic microbes (6, 8). However, several classes of bacterial subcellular compartments are present within many pathogenic bacterial species, including *Mycobacterium tuberculosis*, *Clostridioides difficile*, and several ESKAPE pathogens, among others (3, 4, 11). Recent studies have begun to uncover the importance of subcellular structures to the pathogenicity of these organisms, raising the possibility that these structures may be druggable targets for novel antimicrobial development. In this review, we focus on the contribution to virulence of three major types of subcellular compartments found within bacterial pathogens: (i) bacterial microcompartments, (ii) encapsulin nanocompartments, and (iii) ferrosomes.

## BACTERIAL MICROCOMPARTMENTS

**Fig 1:**
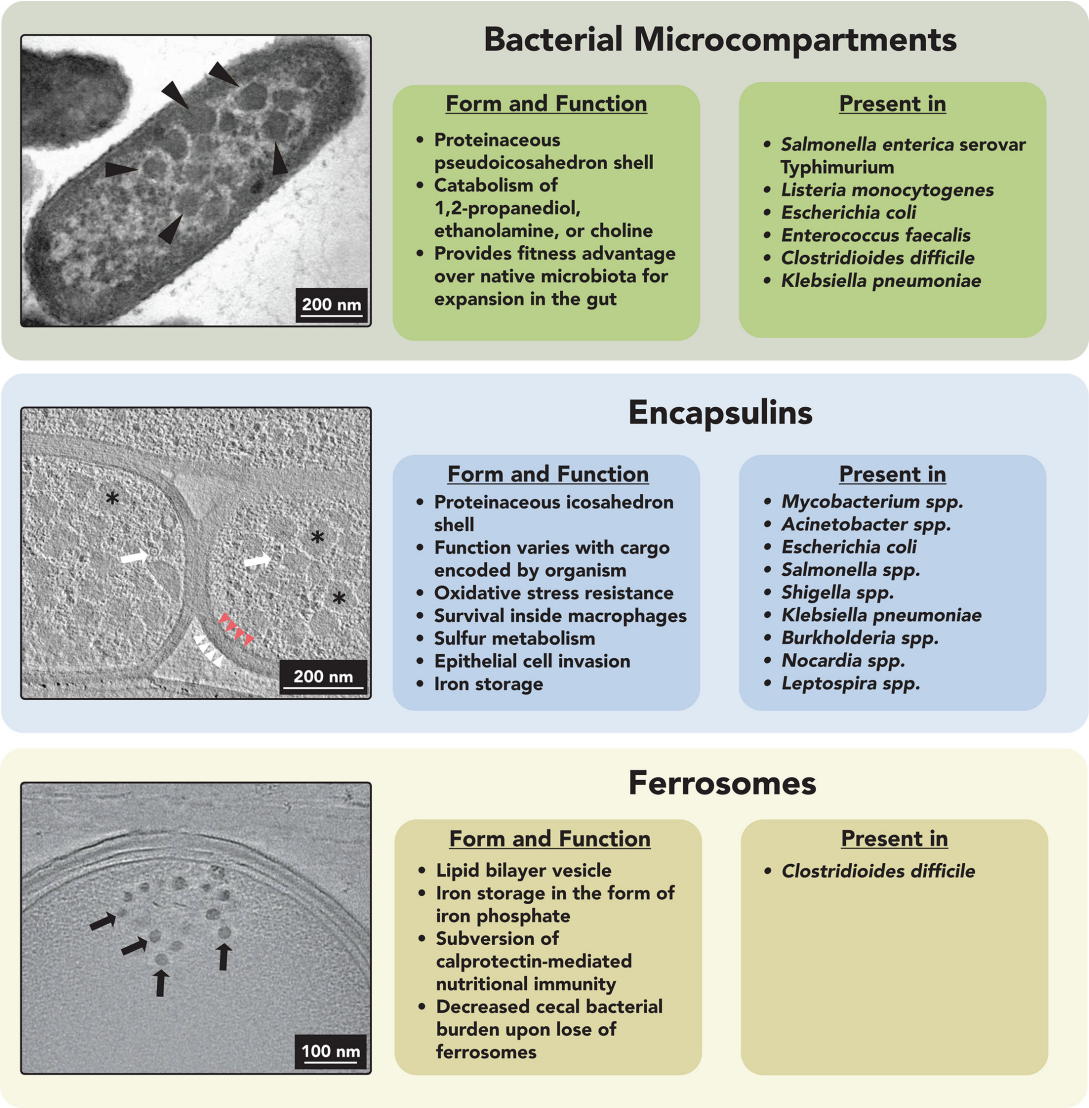
Subcellular compartments found in bacterial pathogens. Representative micrographs, a summary of form and function, and a non-exhaustive list of pathogens containing bacterial microcompartments, encapsulins, and ferrosomes. Black triangles denote bacterial microcompartments. White arrows denote encapsulins. Black arrows denote ferrosomes. Asterisks denote unknown storage granules. Red and white triangles denote inner and outer membranes, respectively. Micrograph of bacterial microcompartments in *S*. Typhimurium (top) is adapted from reference 17. Micrograph of encapsulins in *Mycobacterium marinum* (middle) is adapted from reference 18. Micrograph of ferrosomes in *C. difficile* (bottom) is adapted from reference 19. Micrographs were modified from the originals by cropping and replacement or addition of arrows, triangles, asterisks, and scale bars.

Bacterial microcompartments (BMCs) are self-assembling proteinaceous organelles that encase a system of enzymes that allow for efficient utilization of specific nutrients (9) (Fig. 1). Of the subcellular compartments discussed in this review, BMCs are the most widely distributed, with a total of 45 bacterial phyla predicted to encode at least one BMC system (2). BMC shells are relatively large (100–300 nm in diameter), pseudo-icosahedral compartments composed of several distinct shell protomers (1, 12, 13). Genes encoding shell protomers are typically co-transcribed along with their cargo proteins, which are targeted to the shell interior by a short N- or C-terminal targeting peptide (14–16). The encapsulation of cargo proteins within the semipermeable shell increases catalytic efficiency by spatially organizing enzymes and substrates of a distinct metabolic pathway and protects the cell from possible toxic metabolic intermediates (9). Within pathogenic bacteria, two major BMC classes have been characterized to play a direct role in pathogenesis: the propanediol utilization (Pdu) BMC involved in 1,2-propanediol metabolism and the ethanolamine utilization (Eut) BMC involved in ethanolamine metabolism.

Both the Pdu and Eut BMCs are synthesized by the enteric pathogen *Salmonella enterica* serovar Typhimurium to metabolize host- and microbiota-derived metabolites (20, 21). The encapsulation of the Pdu and Eut enzymes not only increases their catalytic efficiency but also protects the cell from toxic aldehyde intermediates generated by these pathways (9, 22, 23). During infection, *S*. Typhimurium employs virulence factors such as two type III secretion systems to induce intestinal inflammation, leading to the biosynthesis and increased availability of alternate electron acceptors, such as tetrathionate (24–26). The presence of these electron acceptors creates a nutrient niche that allows *S*. Typhimurium to respire microbiota-derived 1,2-propanediol using the microcompartment-enclosed Pdu pathway into 1-propanol and propionate (20). Similarly, ethanolamine, liberated from host phosphatidylethanolamine, becomes another viable carbon source for *S*. Typhimurium and is metabolized by the Eut BMC into ethanol and acetyl phosphate in the presence of tetrathionate produced during inflammation (21, 27). While both pathways require alternative electron acceptors generated during intestinal inflammation, 1,2-propanediol metabolism can occur via anaerobic or aerobic respiration, while ethanolamine utilization only proceeds anaerobically (20, 21). These metabolic pathways allow *S*. Typhimurium to proliferate during colitis through the utilization of nutrients that competing bacteria cannot efficiently leverage.

Highlighting the widespread importance of BMC subcellular compartments during infection, Pdu and Eut BMCs modulate colonization and virulence-associated phenotypes in many other enteric pathogens, including enterohemorrhagic and uropathogenic *Escherichia coli* (EHEC and UPEC), *Listeria monocytogenes*, *Enterococcus faecalis*, *Klebsiella pneumoniae*, and *C. difficile* (28–35). Furthermore, positive associations have been made between ethanolamine utilization and periodontal disease progression, with Eut BMCs encoded by several members of the oral microbiome (36, 37). In addition to the Eut and Pdu BMCs, glycyl radical enzyme-containing microcompartments (GRMs) that catabolize choline (GRM1 and GRM2) and 1,2-propanediol (GRM3, GRM4, GRM5, and GRM6) are abundantly found in members of the gut microbiome and several pathogenic bacterial species (2, 37, 38). It is hypothesized that the GRM microcompartments may offer a similar competitive advantage to bacteria within the microbiome as the Pdu and Eut BMCs, as both choline and 1,2-propanediol are abundant within the gastrointestinal tract (20, 39). Interestingly, the GRM2 locus involved in choline utilization in *E. coli* UPEC 536 is encoded on a pathogenicity island, suggesting involvement in the pathogenesis of the organism (40). However, direct evidence for the role of GRMs in colonization or virulence has yet to be established.

## ENCAPSULIN NANOCOMPARTMENTS

The encapsulin nanocompartment is composed of an icosahedral proteinaceous shell, 20–50 nm in diameter, formed by the oligomerization of a single monomer that shares homology with an HK97 phage-like fold (41, 42) (Fig. 1). Similar to BMCs, encapsulins are typically co-transcribed with a cargo protein localized to the encapsulin lumen by a short targeting peptide or encapsulation domain (41, 43). Encapsulation of cargo enzymes often results in increased catalytic efficiency of the enzymes, and in some cases, the encapsulin compartment is utilized as a nutrient storage compartment (41, 44). However, in contrast to BMCs, encapsulins typically carry only a single cargo protein rather than a set of enzymes of a specific metabolic pathway. Additional accessory proteins are also often co-transcribed within encapsulin-containing operons, with gene neighborhoods and sequence similarity used to divide encapsulins into four major families (3, 41). Encapsulin systems are found in a total of 31 bacterial phyla, with Actinobacteria and Proteobacteria showing the largest abundance of predicted encapsulins. Across this wide range of species, encapsulins have been predicted to encapsulate a diversity of cargo proteins, including iron-mineralization enzymes, DyP peroxidases, cysteine desulfurases (CDs), and isoprenoid biosynthesis enzymes, among others (3, 11, 45). Due to this, the role that these compartments play in bacterial physiology likely varies widely across species. Yet, the exact physiological function of nearly all encapsulin systems remains undefined.

Within bacterial pathogens, the family 1 DyP peroxidase encapsulin is one of the most widespread encapsulin systems and is involved in resistance to oxidative stress through detoxification of reactive oxygen species (3, 41, 46). Bioinformatic approaches predicted this system to be encoded in several enterobacterial pathogens, including *Escherichia*, *Salmonella*, and *Shigella* species, on a mobile genetic element, as well as *Mycobacterium*, *Nocardia*, *Burkholderia*, and *Bordetella* species (3, 11). In *Mycobacterium tuberculosis*, the DyP encapsulin protomer was originally identified as a T-cell antigen in mice and humans and subsequently assigned the name Cfp29 (29 kDa culture filtrate protein) (47, 48). This finding, along with the discovery that Cfp29 is necessary for replication of *M. tuberculosis* in mice, strongly suggested that the Cfp29 DyP encapsulin is involved in the pathogenicity of *M. tuberculosis* (49). A recent study has found that the Cfp29 DyP system is required for full resistance of *M. tuberculosis* to oxidative stress and antibiotics, as well as survival inside of bone marrow-derived macrophages (46).

In addition to the DyP encapsulin system, the family 2A CD encapsulin system is also prevalent in several bacterial pathogens, including *Acinetobacter*, *Mycobacterium*, *Nocardia*, *Burkholderia*, *Klebsiella*, and *Leptospira* species (3). This CD encapsulin is predicted to be involved in sulfur metabolism, and *in vitro* assays have revealed that the *Acinetobacter baumannii* CD encapsulin accumulates elemental sulfur in the encapsulin lumen during sulfur limitation through its CD activity (44, 45). Additionally, in *Mycobacterium leprae*, the encapsulin protomer, termed major membrane protein I (MMPI), elicits a gamma interferon-secreting T-cell response in infected patients (50). While in *Mycobacterium avium*, MMPI is involved in the invasion of epithelial cells via a CDC42-dependent mechanism and contributes to *M. avium* survival in a murine pulmonary infection model, and induces apoptosis in macrophages (51–53). However, at the time of publication of these studies, MMPI was presumed to be a membrane-bound, surface-exposed protein and was not known to form an encapsulin nanocompartment. Therefore, the exact *in vivo* mechanism by which the CD encapsulin nanocompartment elicits these responses remains ambiguous. The physiological role of the CD encapsulin system remains unknown, as well as the fate of the elemental sulfur that is stored within its lumen. Recent publications have hypothesized that the encapsulin-associated rhodanese domain encoded within the CD encapsulin operon may function to mobilize the sulfur pool from the encapsulin lumen (54). However, the biologically relevant sulfur donor and acceptor of this enzyme remain enigmatic. Identification of the final fate of the stored sulfur will enlighten the function of the CD encapsulin system.

## FERROSOMES

Originally characterized in the environmental bacteria *Desulfovibrio magneticus*, ferrosomes are iron-storage microcompartments predicted to exist in many environmental and commensal bacteria (4, 7) (Fig. 1). In contrast to the proteinaceous BMC and encapsulin compartments discussed above, ferrosomes are lipid-bound organelles that are constructed by membrane invagination through an unknown mechanism in response to iron limitation. These vesicles then accumulate iron phosphate within their lumen through the action of a P_1B6_-ATPase, FezB (4, 7, 19). Recent work has discovered that ferrosomes are involved in the virulence of the gastrointestinal pathogen *C. difficile* (19). During infection, the host attempts to sequester vital micronutrient transition metals such as iron, zinc, and manganese away from invading bacteria to limit pathogen proliferation in a process termed nutritional immunity (55). In response to nutritional immunity, *C. difficile* upregulates the machinery necessary to form ferrosomes, allowing the bacteria to sequester and store iron from the host within ferrosomes and subvert nutritional immunity to cause disease (19). Many questions remain unanswered regarding the function of ferrosomes in *C. difficile*. As there exists no homolog of a eukaryotic dynamin-superfamily protein within the *fez* operon, what is the mechanism by which the membrane invaginations are formed? How does formation of ferrosomes under already iron-limited conditions not result in additional cytoplasmic iron-limitation stress? How does the cell access and mobilize the sequestered iron? Can ferrosomes be shared across organisms? Finally, do other ferrosome-like structures exist that sequester metals other than iron?

## CONCLUDING REMARKS

There is increasing evidence that bacterial subcellular structures are intimately involved in bacterial pathogenesis, raising the question of whether these subcellular compartments may prove to be a viable strategy for novel therapeutic development. As an example, an isethionate-metabolizing BMC is essential for intestinal colonization by *Bilophila wadsworthia,* a pathobiont whose expansion within the gut under a high-fat diet has been linked to several pathologies, including inflammation and gut barrier dysfunction (56, 57). Targeting the enzymatic cargo of this BMC to inhibit its activity may lead to control of *B. wadsworthia* within the gut. However, due to the ubiquitous nature of BMCs and encapsulins across bacterial phyla and their abundance within the microbiota, a therapeutic targeting these systems may result in dysbiosis of the microbiome. While BMCs may play important roles in bacterial pathogenesis, a recent study found that depletion of BMC-containing organisms occurs in patients with Parkinson’s disease (58). This finding highlights the important link between changes in the microbiome and certain disease states, complicating therapeutic approaches against BMCs that may lead to changes in the microbiome and undesired outcomes. There remain many unanswered questions regarding the function of these structures and their relevance to virulence in a plethora of clinically important pathogens, as well as the possibility of many yet-to-be identified subcellular structures across the wide breadth of bacterial phyla.
